# Association between metabolic dysfunction-associated steatotic liver disease and cardiovascular disease: New perspectives

**DOI:** 10.1097/MD.0000000000043952

**Published:** 2025-08-15

**Authors:** Qinyi Zhou, Wang Liu, Dan Zhou, Yifang Zhang, Zhaobing Li, Zili Li, Xiaofeng Ma

**Affiliations:** a Department of Cardiology, The Affiliated Nanhua Hospital, Hengyang Medical School, University of South China, Hengyang, Hunan, China; b Institute of Cardiovascular Disease, Key Laboratory for Arteriosclerology of Hunan Province, Hunan International Scientific and Technological Cooperation Base of Arteriosclerotic Disease, University of South China, Hengyang, Hunan,China; c Department of Gastrointestinal Surgery, The Affiliated Nanhua Hospital, Hengyang Medical School, University of South China, Hengyang, Hunan, China.

**Keywords:** cardiovascular disease, metabolic dysfunction-associated fatty liver disease, metabolic dysfunction-associated steatotic liver disease, metabolic syndrome, nonalcoholic fatty liver disease

## Abstract

Metabolic dysfunction-associated steatotic liver disease (MASLD), formerly known as nonalcoholic fatty liver disease (NAFLD), is characterized by systemic insulin resistance and metabolic dysfunction. MASLD/NAFLD elevates the risk of developing cardiovascular disease (CVD). As a quantitative method, bibliometric analysis, illuminates the trajectory of research within a particular field and offers insights into its current state and future directions. In the present study, Citespace (version 6.3.1) was used to comprehensively review the relevant literature for a detailed characterization of the association between MASLD/NAFLD and CVD. This analysis aimed to delineate the historical progression, current research hotspots, and future development trends of MASLD/NAFLD and its relationship with CVD. Our findings highlight a notable surge in research interest in MASLD/NAFLD and CVD over the past 19 years, reflecting an increasing depth of exploration into their interrelationship. In addition to established factors, such as alanine aminotransferase (ALT) and metabolic syndrome, previously overlooked aspects, such as inflammation, gut microbiota, and oxidative stress, have gained significant attention as notable contributors to the pathogenesis of MASLD/NAFLD. By elucidating the intricate association between MASLD/NAFLD and CVD, this study provides prospects for pathophysiological mechanism and preventive strategies for both conditions and provides research insights regarding potential future avenues and focal areas for future investigations.

## 1. Introduction

Metabolic dysfunction-associated steatotic liver disease (MASLD) is defined as hepatic steatosis with at least 1 cardiometabolic risk factor but without any other identifiable cause.^[1]^ The pathophysiological mechanisms underlying MASLD contribute to the development of nonalcoholic fatty liver disease (NAFLD) and is associated with various extrahepatic complications, including cardiovascular disease (CVD) and specific malignancies, such as cirrhosis, liver failure, and hepatocellular carcinoma.^[2–4]^ The definition of MASLD closely parallels that of NAFLD, with approximately 99% of patients with NAFLD meeting the diagnostic criteria for MASLD.^[5]^ In response to this overlap, international experts have proposed the renaming of NAFLD as metabolic dysfunction-associated fatty liver disease (MAFLD), with nonalcoholic steatohepatitis being renamed metabolic dysfunction-related steatohepatitis. Nowadays, the name chosen to supersed NAFLD was MASLD.^[1]^ MAFLD not only demonstrates a significantly associated with increased CVD risk but also demonstrates superior predictive performance for incident CVD.^[6]^ The leading cause of mortality in patients with NAFLD is CVD, and NAFLD is closely associated with an increased risk of major CVD events and other cardiac complications including as cardiomyopathy, cardiac valve calcification, and arrhythmias.^[7]^ A retrospective cross-sectional study demonstrated comparable prevalence rates between MASLD and NAFLD. However, MASLD exhibits superior clinical utility in identifying patients at heightened risk of metabolic derangements or cardiovascular events.^[8]^ Notably, MASLD has been linked to a spectrum of cardiovascular complications. Moon et al found that the risk of sudden CVD occurrence is increased in patients with MASLD, and alcohol consumption is a risk factor for cardiometabolic disorders and promotes the development of CVD.^[9]^ Therefore, necessitating a comprehensive reassessment of cardiovascular risk in patients with MASLD. This reassessment should encompass diverse factors, including age, sex, family history, ethnicity, smoking status, alcohol consumption, lipid levels, body mass index, blood pressure, and the presence of diabetes, alongside a consideration of other comorbidities unrelated to MASLD/NAFLD and yet associated with heightened cardiovascular risk, such as rheumatoid arthritis, and treatments for severe mental illness.^[10–17]^ Therefore, adopting a holistic approach that incorporates these considerations is imperative when evaluating cardiovascular risk in individuals with MASLD/NAFLD.

We conducted an exhaustive search for articles published in the past 19 years that are included in the Web of Science Core Collection (WoSCC) to elucidate the association between MASLD/NAFLD and CVD. Our search included the search terms “MASLD,” “NAFLD,” and “MAFLD,” and yielded a total of 3115 articles, encompassing diverse publication, such as experimental study articles, conference reports, reviews, letters, books, and online publications. Because of the sheer volume of the literature identified by our literature search, summarizing the key findings from these articles was an intensive task. Therefore, we used Citespace software in this study to analyze and present the pivotal points and emerging research trends within this field of research. Our primary objective was to highlight the articles that have significantly contributed to unraveling the link between MASLD/NAFLD and CVD, while also identifying current areas of intense research. In addition, our analysis aimed to illuminate future research directions and novel areas of interest, thereby providing innovative ideas and methodologies for clinical diagnosis, treatment strategies, and prevention strategies targeting MASLD/NAFLD and CVD.

## 2. Materials and methods

### 2.1. Literature retrieval

WoSCC comprises a vast array of scholarly journals, conference papers, and academic literature spanning multiple disciplines. It serves as a valuable resource for researchers conducting comprehensive literature searches and citation analyses. In the present study, we conducted a meticulous screening process while searching the WoSCC, resulting in the identification of 3115 articles exploring the relationship between MASLD/NAFLD and CVD over a 19-year period. MASLD was referred to as NAFLD in the early years of the period covered by the literature, and all variations of MASLD names were included in the search criteria settings to ensure inclusivity and thoroughness.

### 2.2. Bibliometrics and bibliometrics analysis tools

Bibliometrics is a scientific discipline that examines the characteristics and volume of scientific literature, aiming to quantify and analyze various aspects of research output, including citations, impact factor values, and other relevant indicators. Its primary objective is to elucidate patterns in research development, identify interdisciplinary connections, and illuminate collaborative relationships within academic research.

Citespace is a quantitative analysis tool designed for analyzing scientific literature, facilitating the visual representation of correlations between publications, research hotspots, and discipline-specific research development trends. When examining literature connections, dense clusters typically signify established research domains, whereas isolated nodes may denote emerging research directions or peripheral fields. The size and color variations of nodes correspond to the significance or impact of a scientific article; notably, heavily cited articles are often depicted as larger or more prominent nodes within the network. Furthermore, Citespace software incorporates keywords extracted from the literature to pinpoint research hotspots and critical areas of study. The “#” symbol is used in the text to mark each of the different keyword clusters. During the Citespace software analysis, the node selection method was configured using the *g*-index, with the corresponding *K*-value set to the maximum permissible value within the algorithm’s operational parameters.

## 3. Results

### 3.1. Relevance of MASLD and CVD research areas

**Figure 1.**
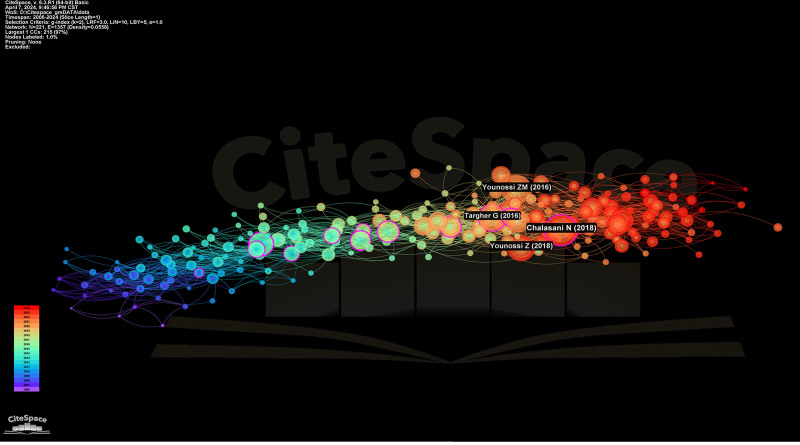
Citation co-occurrence network. The years 2006 to 2024 are shown by color bars.

We used Citespace software to analyze a comprehensive dataset comprising 3115 literature sources published during 2006 to 2024, focusing on exploring the association between MASLD/NAFLD and CVD. The analysis outcomes sre depicted visually through a citation co-citation graph (Fig. 1), showing 221 nodes and 1357 links.

Our analysis of the initial period covered by the studies (2006–2010), represented by blue-purple nodes, primarily concentrated on elucidating the role of metabolic syndrome (MetS) and insulin resistance in mediating the relationship between MASLD/NAFLD and CVD. The observations from this period laid the groundwork for subsequent investigations into this association.

During the intermediate period (2011–2018) covered by the literature, denoted by yellow-green nodes, with purple rings signifying high intermediate centrality, greater attention was directed toward identifying risk factors influencing both MAFLD/NAFLD and CVD. Notably, studies by researchers such as Chalasani et al,^[18]^ Younossi et al,^[19]^ Younossi et al,^[20]^ and Targher et al^[21]^ garnered significant citation frequencies, indicating their noteworthy contributions to understanding the intricate relationship between MASLD/NAFLD and CVD.

In the later period covered by the studies (2019–2024), represented by orange-red nodes exhibiting increased density and outward branching, a discernible surge in interest toward exploring diverse aspects concerning the association between MASLD/NAFLD and CVD was observed, reflecting a broader scope of research inquiries and potential avenues for further investigation.

**Figure 2.**
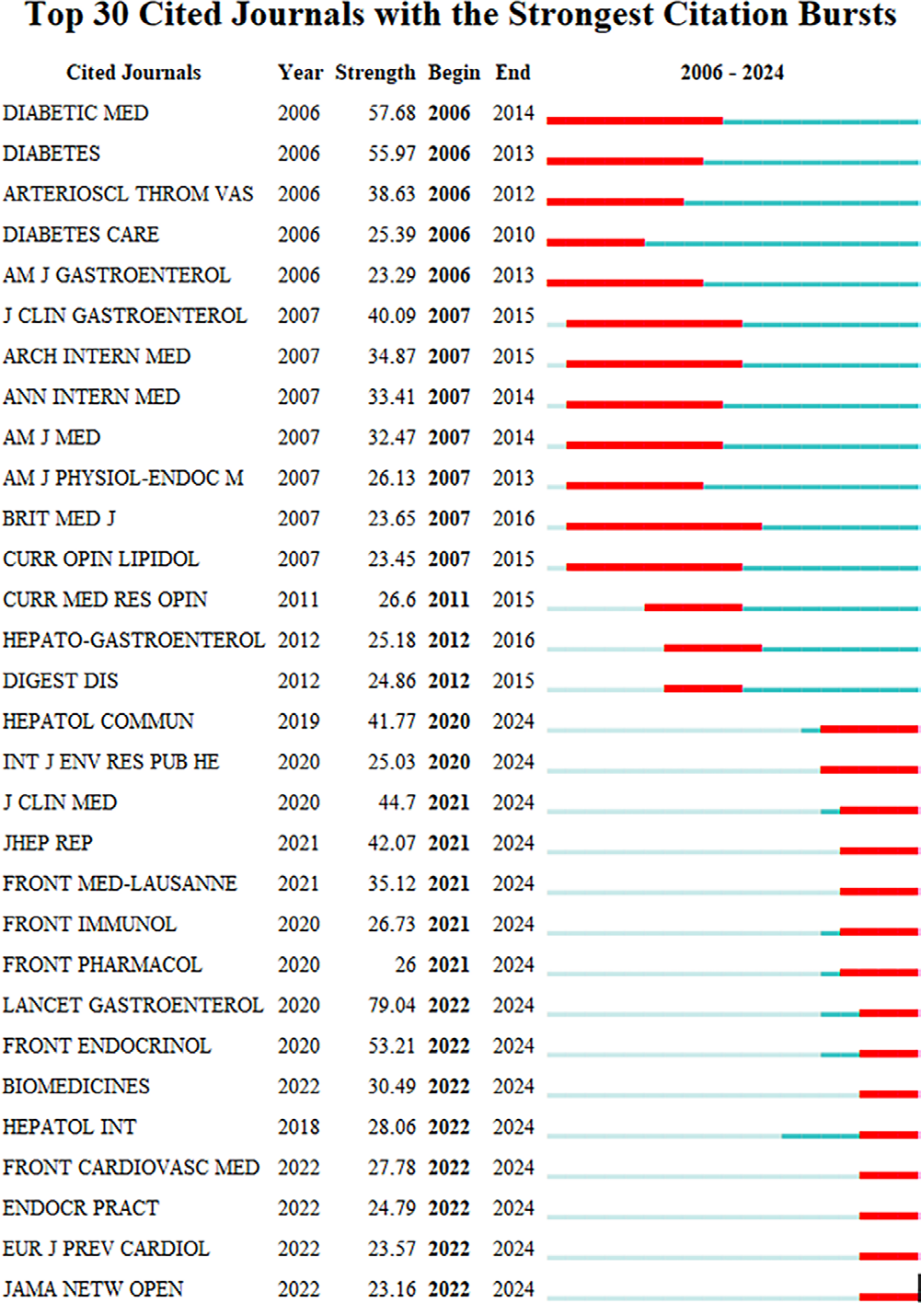
Top 30 cited journals.

We conducted an exhaustive analysis of the literature pertaining to the correlation between MASLD/NAFLD and CVD spanning 2006 to 2024. Through this analysis, we identified and ranked the top 30 highly cited journals (Fig. 2). In the figure, the red line segments denote periods of significant citation bursts, indicating both the initiating and concluding years of each burst. Notably, the journal named DIABETIC MED exhibited a sudden surge in citations during 2006 to 2014, with the outbreak intensity peaking at 57.68.

### 3.2. Burst maps of cited literature

The Citespace breaking chart is used to identify sudden surges in the number of citations within a specific timeframe, indicating significant research achievements or theoretical breakthroughs in a particular field, as well as influential literature. The emergence of burst maps for cited literature can help elucidate the reasons behind the sudden attention and widespread citation of research articles with certain results or theories. This may be attributed to their substantial impact on other studies within the field or their ability to address crucial problems, thereby attracting significant attention from the academic community. Consequently, burst maps of cited literature serve as valuable tools for researchers to discern important research outcomes and theories, facilitating an understanding of current research trends and cutting edge directions within a specific field.

**Figure 3.**
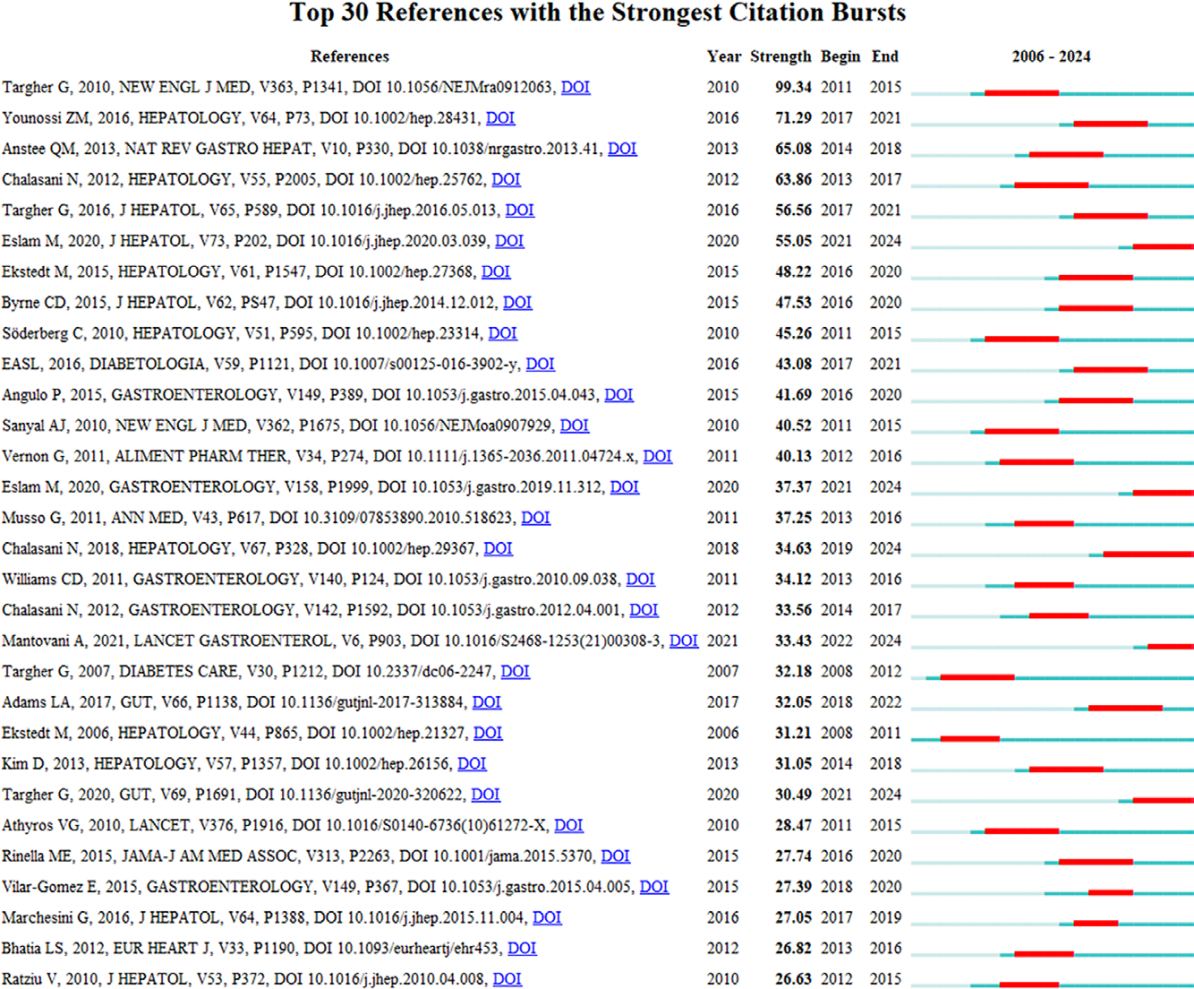
Top 30 citations (sorted by burst intensity).

Based on our analysis of 3115 literature sources spanning 2006 to 2024, we compiled a list of the top 30 highly cited articles based on burst intensity (Fig. 3). The article by Targher is the most frequently cited publication, with a notable burst intensity of 99.34.^[22]^ This article delves into the risk factors associated with CVD in individuals diagnosed with NAFLD. The findings reported therein suggest that patients with NAFLD are more susceptible to developing cardiovascular complications, potentially attributed to inflammation and metabolic disorders triggered by fatty liver disease. Further, preventive measures and treatment strategies for NAFLD, such as dietary modifications, increased physical activity, and medication are also discussed.^[22]^ As the foremost influential article establishing a link between NAFLD and CVD, it serves as a foundational reference for subsequent research endeavors.^[22]^

The study by Younossi, ranking second in terms of burst intensity, with a notable burst intensity of 71.2, gained significant attention during 2017 to 2021.^[19]^ This study primarily focused on global epidemiology pertaining to NAFLD. Through comprehensive meta-analysis, data regarding prevalence rates, incidence rates, prognostic outcomes related to NAFLD along with its association with CVD were summarized. Individuals afflicted by NAFLD often exhibit obesity, insulin resistance or type-2 diabetes mellitus comorbidity alongside dyslipidaemia, hypertriglyceridemia and hypertension; these are all recognized risk factors for cardiovascular ailments. Cardiovascular-related mortality is one of the leading causes among patients suffering from NAFLD.^[23–25]^ Thus, it can be inferred that an inherent association between NAFLD and CVD exists, which highlights the potential risks involved. This insight provides valuable direction for future investigations on the relationship between MASLD/NAFLD and CVD.

**Figure 4.**
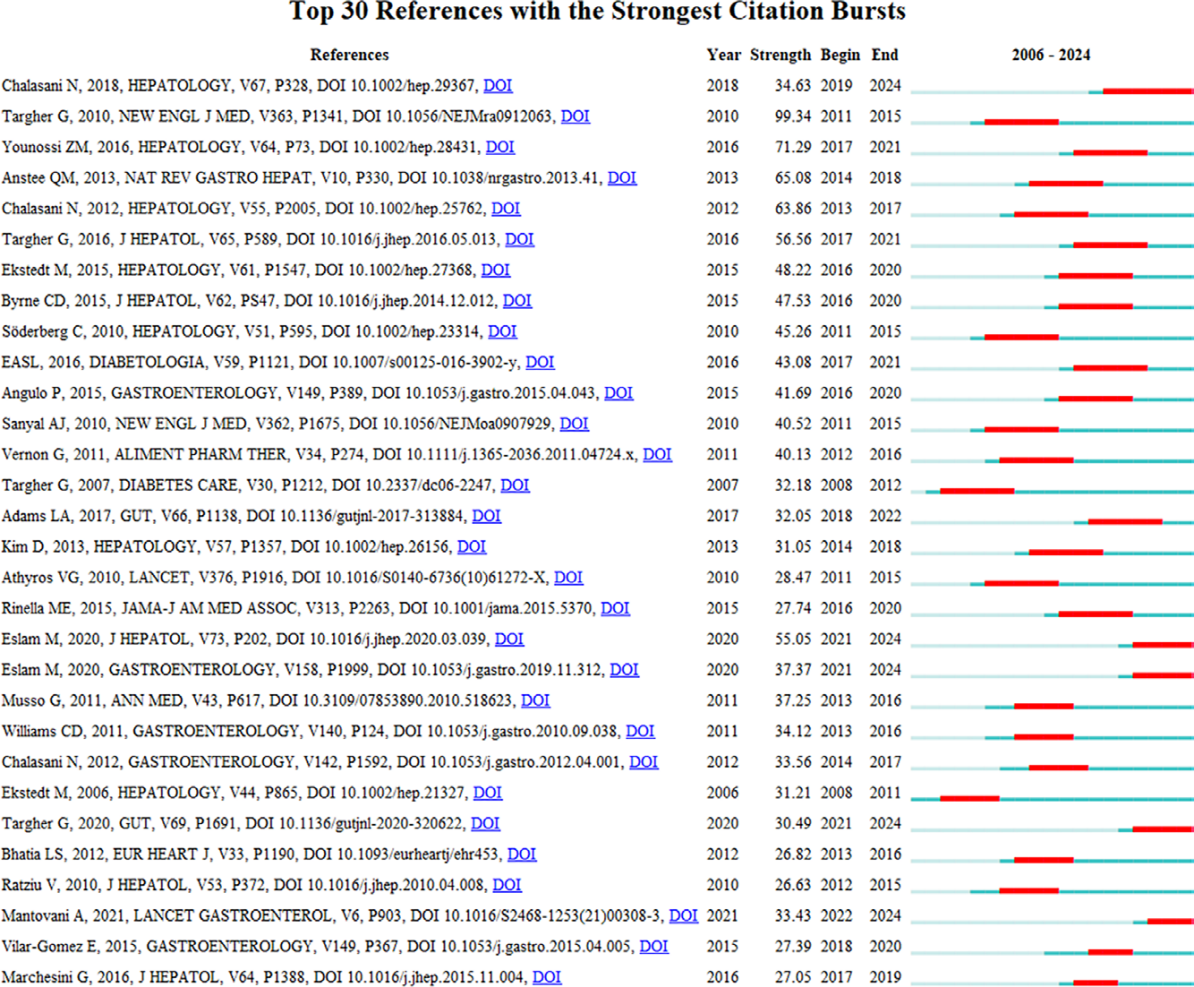
Top 30 citations (sorted by burst duration).

Based on the duration of the burst in cited literature, we classified them further (Fig. 4). Five publications had consistently attracted attention. Authored by Chalasani et al,^[18]^ Eslam et al,^[26,27]^ Targher et al,^[22]^ and Mantovani et al,^[28]^ these research articles provide comprehensive insights into the diagnosis, treatment, and prevention of NAFLD. They propose a novel definition for NAFLD and extensively discuss its diagnostic criteria, pathological features, clinical manifestations, and treatment strategies. Moreover, they advocate for sub-phenotyping and stratification of patients using precise genetic, anthropometric, and metabolic phenotyping methods to enable personalized risk prediction and prevention strategies, thereby enhancing clinical trial design. In addition, these studies explored the pathophysiological mechanisms linking NAFLD with CVD and summarized pharmacological treatments for NAFLD that may positively or negatively impact CVD risk. Notably, a systematic review and meta-analysis comparing individuals with NAFLD and controls without NAFLD revealed an increased risk of fatal or nonfatal cardiovascular events in patients with NAFLD. Further, this analysis established a positive correlation between the severity of NAFLD and likelihood of experiencing cardiovascular events.^[7,18,26–28]^ Notably, this emphasis on investigating the association between MASLD/NAFLD and CVD reflects recent research trends.

### 3.3. Keyword burst

Keyword burst analysis facilitates the identification of research hotspots and emerging trends within specific fields, enabling researchers to comprehend the focal points and developmental trajectories of these domains. Moreover, it enables researchers to grasp the prevailing issues in their respective fields. Furthermore, keyword burst analysis can uncover nascent research directions that may have been overlooked or may have received insufficient attention, thereby providing researchers with opportunities to broaden their scholarly interests and understanding.

**Figure 5.**
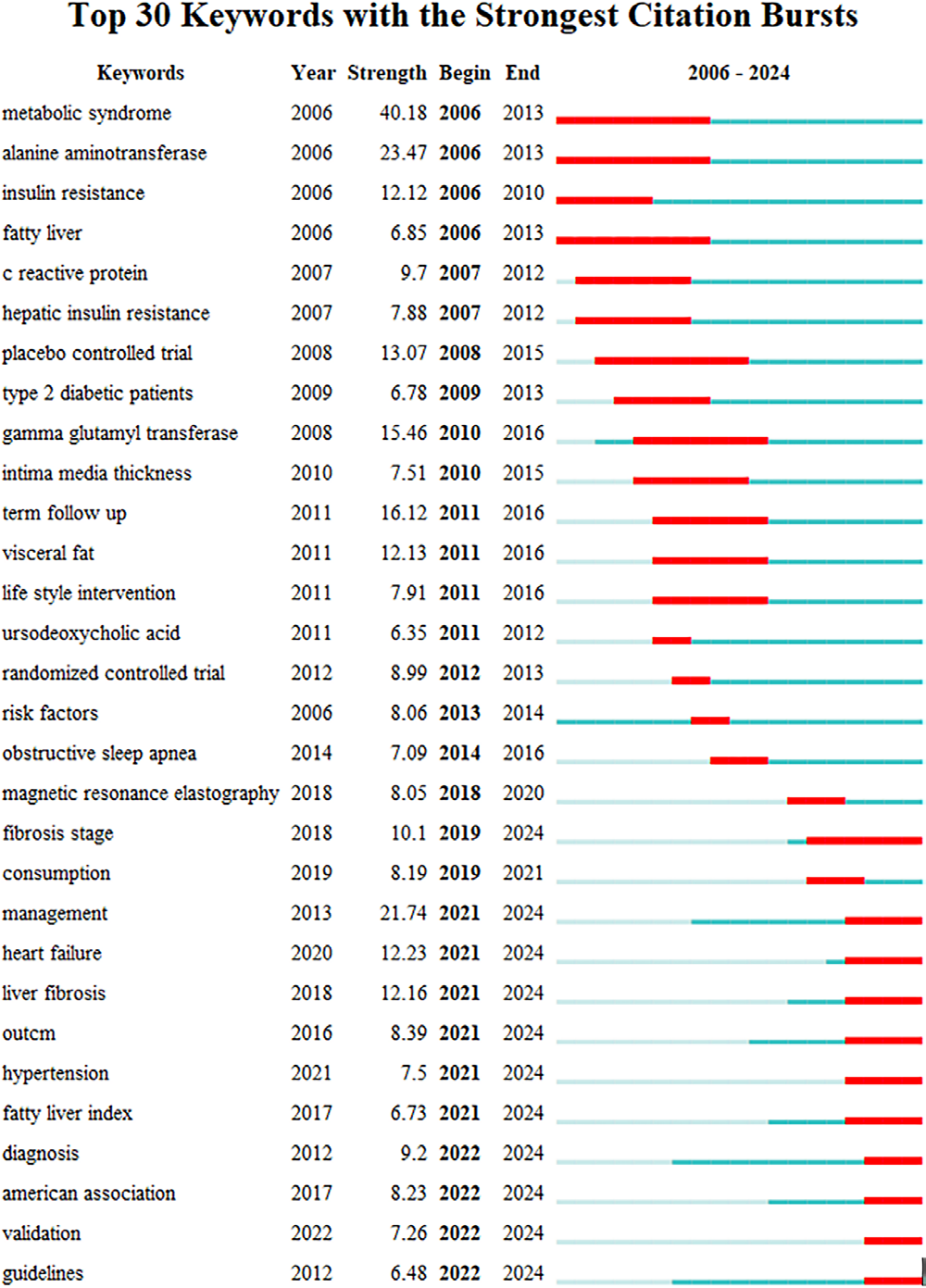
Top 30 keywords (sorted by first burst).

To delve deeper into the correlation between MASLD/NAFLD and CVD, we conducted an analysis of keywords appearing in relevant articles spanning 2006 to 2024, categorizing them based on the year of their initial burst and compiling the top 30 burst keywords (Fig. 5) to assess recent research focal points.

MetS emerges as the earliest and most intense keyword burst, with an intensity of 40.18, thus highlighting its pivotal role in MASLD/NAFLD and CVD research. Keywords appearing during 2006 to 2013, including MetS, metabolic disorder disease, and symptoms, such as obesity, high blood pressure, high blood sugar, and blood fat, are recurrent themes. MetS encompasses abdominal obesity, hypertension, hyperglycemia, dyslipidaemia, and insulin resistance, and these are closely connected to CVD incidence. Among patients with NAFLD, MetS incidence has been reported at 25.4 per 1000 person-years, akin to 24.77 per 1000 person-years for CVD, thereby implicating MetS as a key driver of CVD development, particularly in patients with NAFLD.^[29]^ Investigating the role of the MetS in the interplay between MASLD/NAFLD and CVD has been a focal point of scientific inquiry during the initial and intermediate periods covered by the literature.

During the same period, the keyword of alanine aminotransferase (ALT) also demonstrated a notable burst, with an intensity of 23.47. ALT, a liver enzyme indicative of liver function and the extent of hepatic damage, was elevated in MASLD/NAFLD owing to liver fat accumulation, suggesting its potential as a MASLD/NAFLD marker. The association of ALT with CVD has also been explored, with elevated ALT levels linked to increased cardiovascular risk. However, the precise mechanism remains elusive, necessitating further investigation to delineate the role of ALT in CVD.^[30–33]^ Research elucidating the interplay between MASLD/NAFLD and CVD has remained a primary focus over the years.

To investigate the contemporary research landscape concerning the interplay of MASLD/NAFLD and CVD, we focused on emerging keyword bursts after 2021. The gradual appearance of 4 keywords in the pertinent literature, namely heart failure, liver fibrosis, health management, and hypertension (Fig. 5), is noteworthy. This suggests a recent shift in focus within MASLD/NAFLD and CVD research, with novel avenues of inquiry gaining traction. Therefore, it is imperative to closely monitor forthcoming studies for deeper insights and understanding.

### 3.4. Keyword clustering

**Figure 6.**
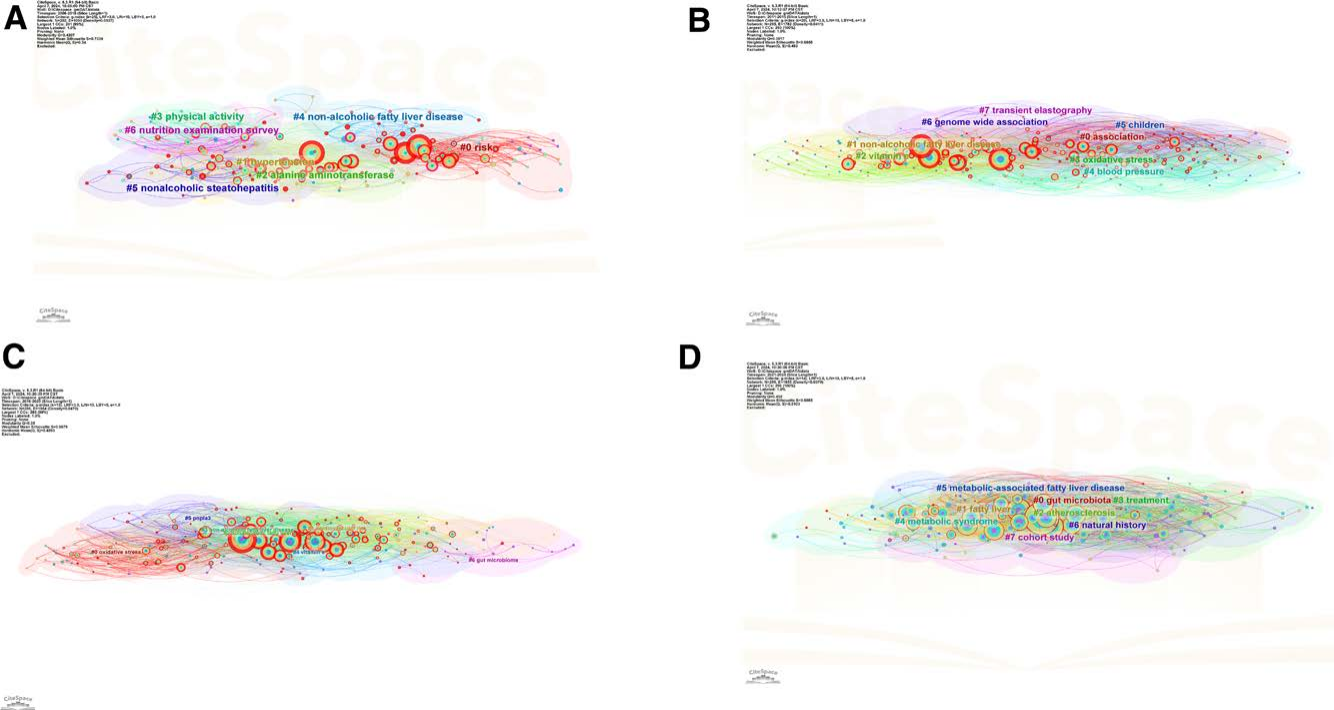
(A) Keyword clustering for the first stage (2006–2010), (B) keyword clustering for the second stage (2011–2015), (C) keyword clustering for the third stage (2016–2020), and (D) keyword clustering for the fourth stage (2021–2024).

We used keyword co-occurrence network analysis to cluster keywords, thereby revealing the interconnectedness between keywords based on their co-occurrence in relevant literature. The resulting clusters of keywords facilitated an examination of the prominent areas related to the association between MASLD/NAFLD and CVD. We categorized the time period of 2006 to 2010 as the first stage, 2011 to 2015 as the second stage, 2016 to 2020 as the third stage, and 2021 to 2024 as the fourth stage. The keyword cluster analysis diagram is shown in Figure 6.

In the first stage, there were 202 nodes and 1090 links that were categorized into 7 clusters including #0 risk, #1 hypertension, and #2 ALT (Fig. 6A). In the second stage, we observed 295 nodes and 1782 links, forming 8 clusters, including #0 association, #1 NAFLD, and #2 vitamin E (Fig. 6B). During the third stage, there were 286 nodes and 1954 links, forming 6 clusters, including #0 oxidative stress, #1 cardiovascular risk, and #2 NAFLD (Fig. 6C). Lastly, in the fourth stage, a total of 296 nodes and 1655 links constituted 8 distinct clusters, including #0 gut microbiota, #1 fatty liver, and #2 atherosclerosis (Fig. 6D). In the first 2 stages, research on ALT, MetS, and insulin resistance received significant attention, providing a solid foundation for research on MASLD/NAFLD and CVD. In recent years, several new keyword clusters have emerged, such as #oxidative stress, #gut microbiota, and #adipose tissue inflammation. These newly emerged keyword clusters represent the current research hotspot.

Liu et al demonstrated that the aspartate aminotransferase (AST) to ALT ratio is associated with increased risks of all-cause mortality and cardiovascular mortality.^[34,35]^ ALT was somewhat inversely associated with coronary heart disease.^[36]^ However, the precise pathophysiological mechanism underlying the impact of ALT on CVD remains to be fully elucidated. MetS is a clinical constellation characterized by a cluster of metabolic abnormalities, including hypertension, central obesity, insulin resistance, and atherogenic dyslipidemia. MetS is strongly associated with a significantly elevated risk of developing atherosclerotic CVD.^[37]^ Insulin resistance, neurohormonal activation, and chronic inflammation appear to be the main players in the initiation, progression, and transition of MetS to CVD.^[38]^

Oxidative stress is recognized as a major underlying component in the pathophysiology of several diseases, including MetS, atherosclerosis, and NAFLD.^[39–41]^ Oxidative stress can reduce the bioavailability of nitric oxide and cause endothelial cell dysfunction. In addition, it can trigger inflammatory responses and mitochondrial dysfunction, further impacting endothelial cell function. Therefore, oxidative stress can lead to endothelial dysfunction in both CVD and MASLD/NAFLD, thereby promoting the development of the disease.^[42–45]^ Furthermore, oxidative stress can exacerbate the pathological progression of MASLD/NAFLD and CVD through mechanisms including mitochondrial dysfunction, inflammatory responses, excessive accumulation of reactive oxygen and nitrogen species, and aberrant lipid deposition.^[46,47]^

Studies have shown that alterations in gut microbiota are associated with the development of obesity and insulin resistance, and microbial transplantation can transmit the obese state of the host. Disruption of gut microbiota may also promote obesity at an early stage.^[48–50]^ In addition, presence of bacterial DNA in human atherosclerotic plaques have also been reported, suggesting that gut floras are involved in atherosclerosis.^[51,52]^ Alterations in gut microbiota are closely interrelated with the development of MASLD/NAFLD and CVD through common pathways, including carbohydrate metabolism, lipid metabolism, and inflammation.^[53,54]^ Gut microbiota-derived metabolites, such as trimethylamine N-oxide, fatty acids, and phenylacetylglutamine, play a pivotal role in the pathogenesis of MASLD/NAFLD and CVD by modulating hepatic lipid accumulation and foam cell formation.^[55–57]^

Adipose tissue inflammation is caused by an abnormal proliferation of adipocytes and infiltration of inflammatory cells adjacent to adipocytes. In MASLD/NAFLD, adipose tissue inflammation can lead to the death and rupture of fat cells, releasing large amounts of fatty acids and cytokines, such as tumor necrosis factor-α and interleukin-6, which can further promote liver inflammation and fibrosis. Adipose tissue inflammation can also lead to insulin resistance and the development of a fatty liver by affecting liver insulin signaling pathways and lipid metabolism.^[58–60]^ In addition, adipose tissue inflammation can lead to endothelial dysfunction and atherosclerosis. The release of inflammatory cells and cytokines can cause endothelial cell injury and trigger inflammatory response in the blood vessel wall, promoting the formation of atherosclerotic plaques.^[61–64]^ Moreover, dysregulated expression of inflammation-related miRNAs promotes adipose tissue inflammation and insulin resistance, thereby exacerbating metabolic homeostasis disruption. In CVD, aberrant miRNA expression alters inflammatory responses, impairs endothelial function, and induces cardiac remodeling, thereby driving disease progression. In MASLD/NAFLD, inflammation-associated miRNAs are mechanistically involved in mediating hepatic inflammation, lipid deposition, and fibrotic processes.^[65]^

In conclusion, factors including ALT, MetS, gut microbiota, oxidative stress, and adipose tissue inflammation collectively influence the progression of MASLD/NAFLD and CVD. Shared risk factors and pathophysiological mechanisms between MASLD/NAFLD and CVD require further elucidation and in-depth investigation. Critical unresolved questions include: Through which molecular pathways does ALT regulate CVD progression? Given the increasingly defined role of gut microbiota and their metabolites in MASLD/NAFLD and CVD pathogenesis, can probiotics or prebiotics be developed for their prevention or treatment? Identifying cross-disease-specific therapeutic targets common to both MASLD/NAFLD and CVD represents a critical mission in basic medical research.

### 3.5. Timeline visualization

**Figure 7.**
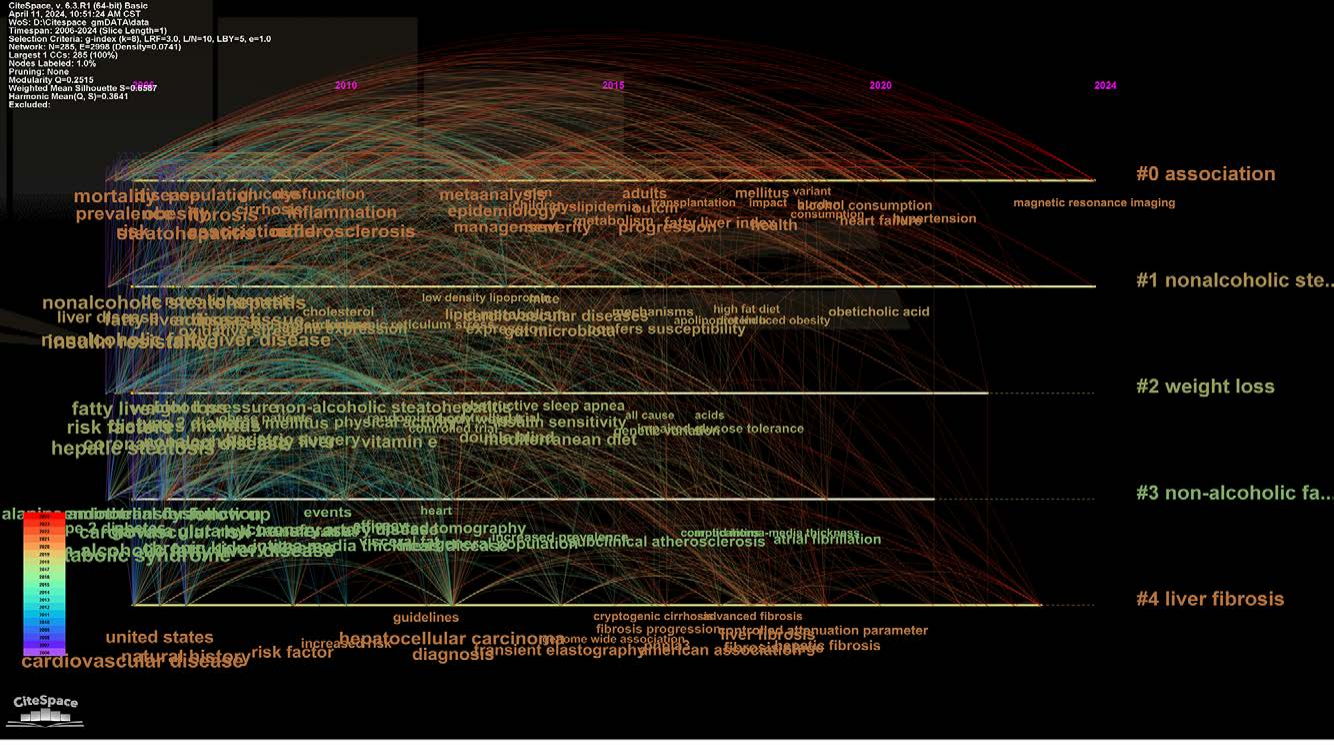
The keyword clustering timeline for the whole stage.

**Figure 8.**
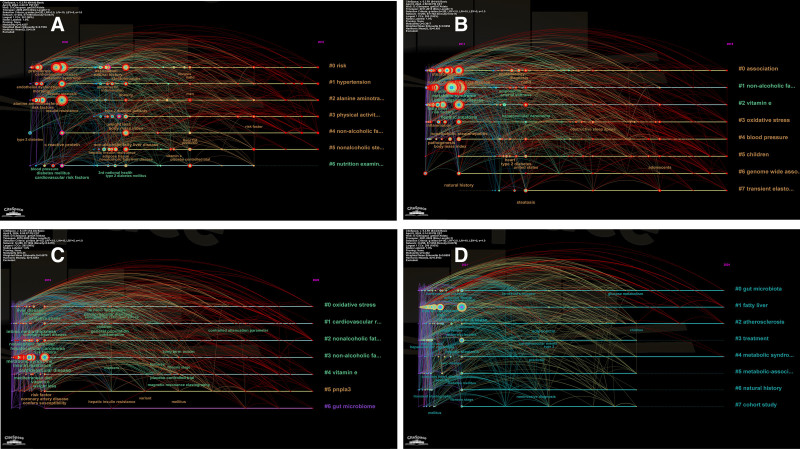
(A) The keyword clustering timeline for the first stage (2006–2010), (B) the keyword clustering timeline for the second stage (2011–2015), (C) the keyword clustering timeline for the third stage (2016–2020), and (D) the keyword clustering timeline for the fourth stage (2021–2024).

Timeline visualization serves as a valuable tool for researchers to track the development of specific research areas over time, capturing shifts in focus, key discoveries, and evolving trends. Initially, we constructed a comprehensive timeline spanning 2006 to 2024 (Fig. 7), providing an overarching view of changes in research hotspots. Subsequently, we segmented this timeline into 4 distinct stages, each representing a defined period (Fig. 8). To facilitate clarity in observation, we have presented the corresponding keyword clusters for each stage in Tables 1–4.

**Table 1:** Keyword clusters for the first stage (2006–2010).

Cluster ID	Cluster keywords	Representative keywords
0	Risk	Metabolic syndrome; nonalcoholic fatty liver disease; fatty liver; risk factor; nonalcoholic steatohepatitis cardiovascular disease; glycemic load; liver tumors; mellitus; body mass
1	Hypertension	Nonalcoholic fatty liver disease; cardiovascular disease; metabolic syndrome; cohort; diabetes mellitus metabolic syndrome; hepatic steatosis; incretin mimetics; metabolic syndromne; nonalcoholic fatty liver disease
2	Alanine aminotransferase	Metabolic syndrome; insulin resistance; cardiovascular disease; nonalcoholic fatty liver disease; systems biology nonalcoholic fatty liver disease; risk factors; systems biology; CVD risk factors; transcription factors
3	Physical activity	Adiposity rebound; behavior modification; eating frequency; school performance; parental BMI childhood obesity; abdominal fat; visceral fat; behavior modification; physical education
4	Nonalcoholic fatty liver disease	Nonalcoholic fatty liver disease; type-2 diabetes; practice guidelines; metabolic risk factors; C-reactive protein metabolic syndrome; cardiovascular disease; risk factors; nonalcoholic steatohepatitis; carotid artery intima-media thickness
5	Nonalcoholic steatohepatitis	Insulin resistance; nonalcoholic fatty liver disease; nonalcoholic steatohepatitis; beta cell; HepG2 cells nonalcoholic fatty liver disease; nonalcoholic steatohepatitis; beta cell; de novo; lipid homeostasis
6	Nutrition examination survey	Insulin resistance; treatment panel; CVD risk factors; nonalcoholic fatty liver disease; obstructive sleep cardiovascular risk; metabolic syndrome; body mass; nutrition examination; United States

BMI = body mass index, CVD = cardiovascular disease.

**Table 2:** Keyword clusters for the second stage (2011–2015).

Cluster ID	Cluster keywords	Representative keywords
0	Association	Nonalcoholic fatty liver disease; cholesterol-lowering drugs; all-cause mortality; non-cardiovascular mortality; coronary artery disease metabolic syndrome; liver disease; coronary artery disease; arterial stiffness; metabolic control
1	Nonalcoholic fatty liver disease	Nonalcoholic fatty liver disease; metabolic syndrome; alanine aminotransferase; nonalcoholic steatohepatitis; aspartate aminotransferase nonalcoholic fatty liver disease; omega-3 fatty acids; fatty acids; controlled trial; arterial stiffness
2	Vitamin E	Fatty liver; metabolic syndrome; nonalcoholic steatohepatitis; hepatic steatosis; growth hormone nonalcoholic fatty liver disease; nonalcoholic steatohepatitis; clinical trials; coronary artery disease; obesity fatty liver
3	Oxidative stress	Nonalcoholic fatty liver disease; metabolic syndrome; lipid metabolism; hepatic metabolic pathways; aerobic capacity nonalcoholic fatty liver disease; insulin resistance; gut microbiota; coronary artery disease; hepatic metabolic pathways
4	Blood pressure	Metabolic syndrome; nonalcoholic fatty liver disease; hepatic metabolic pathways; antioxidant status; aerobic capacity nonalcoholic fatty liver disease; novo lipogenesis; hepatic metabolic pathways; obstructive sleep; body mass
5	Children	Nonalcoholic fatty liver disease; carotid intima-media thickness; ventricular hypertrophy; liver cirrhosis; systolic diameter nonalcoholic fatty liver disease; high-density lipoprotein cholesterol; cardiovascular disease risk; nonalcoholic steatohepatitis; arterial stiffness
6	Genome wide association	NAFLD; genome wide association; TM6SF2 variant; confers susceptibility; PNPLA3 metabolic syndrome; risk factors; carbohydrate; steatosis; saturated fat
7	Transient elastography	Nonalcoholic fatty liver disease; mitochondrial dysfunction; nonalcoholic steatohepatitis; isobutyl-isonitrile liver scintigraphy; coronary artery disease nonalcoholic fatty liver disease; chronic kidney disease; diabetes mellitus; coronary computed tomography angiography; coronary artery disease

NAFLD = nonalcoholic fatty liver disease.

**Table 3:** Keyword clusters for the third stage (2016–2020).

Cluster ID	Cluster keywords	Representative keywords
0	Oxidative stress	Nonalcoholic fatty liver disease; oxidative stress; endothelium dysfunction; arterial stiffness; fatty pancreas nonalcoholic fatty liver disease; natural modulators; diabetes mellitus; hepatocellular carcinoma; arterial stiffness
1	Cardiovascular risk	Nonalcoholic fatty liver disease; metabolic syndrome; arterial stiffness; carotid intima-media thickness; postmenopausal women nonalcoholic fatty liver disease; cardiac arrhythmias; alcoholic liver disease; endocrine system; virus-associated fatty liver disease
2	Nonalcoholic fatty liver	Nonalcoholic fatty liver disease; hepatocellular carcinoma; nonalcoholic steatohepatitis; intrahepatic cholangiocarcinoma; bidirectional relationship nonalcoholic fatty liver disease; insulin resistance; fatty liver disease; virus-associated fatty liver disease; arterial stiffness
3	Nonalcoholic fatty liver disease	Nonalcoholic fatty liver disease; metabolic syndrome; carotid intima-media thickness; arterial stiffness; flow-mediated dilatation nonalcoholic fatty liver disease; fatty liver index; glomerular filtration rate; chronic kidney disease; controlled attenuation parameter
4	Vitamin E	Nonalcoholic fatty liver disease; nonalcoholic steatohepatitis; magnetic resonance; chronic renal failure; NAFLD activity score nonalcoholic fatty liver disease; diabetes mellitus; protein-coupled bile acid receptor-1; gallstone disease; magnetic resonance
5	PNPLA3	Nonalcoholic fatty liver disease; metabolic syndrome; diabetes mellitus; cardiovascular diseases; dietary supplements cardiovascular disease; arterial stiffness; aortic flow propagation velocity; systems biology; coronary artery disease
6	Gut microbiome	Insulin resistance; nonalcoholic fatty liver disease; cardiovascular effect; multisystemic disease; magnetic resonance imaging gut microbiome; mediterranean diet; western diet; vegetarian diet; liver steatosis

NAFLD = nonalcoholic fatty liver disease.

**Table 4:** Keyword clusters for the fourth stage (2021–2024).

Cluster ID	Cluster keywords	Representative keywords
0	Gut microbiota	Insulin resistance; cardiovascular disease; nonalcoholic fatty liver disease; ubiquitin-specific protease; adipose tissue inflammation metabolic syndrome; oxidative stress; mitochondria dysfunction; milk peptide; adipose tissue inflammation
1	Fatty liver	Nonalcoholic fatty liver disease; cardiovascular disease; diabetes mellitus; coronary artery disease; arterial stiffness nonalcoholic fatty liver disease; hepatic steatosis; systematic review; impaired lung function; weekend catch-up sleep
2	Atherosclerosis	Nonalcoholic fatty liver disease; nonalcoholic steatohepatitis; systolic dysfunction; vascular dysfunction; fat score cardiovascular disease; metabolic dysfunction-associated fatty liver disease; cardiac remodeling; type 2 diabetes; gene polymorphisms
3	Treatment	Nonalcoholic fatty liver disease; nonalcoholic steatohepatitis; systems biology; areca catechu; sodium-glucose cotransporter nonalcoholic fatty liver disease; liver fibrosis; systems biology; areca catechu; sodium-glucose co-transporter
4	Metabolic syndrome	Nonalcoholic fatty liver disease; computed tomography; risk stratification; coronary artery calcium; NAFLD activity score nonalcoholic fatty liver disease; fatty liver; liver disease; healthy patients; metabolic syndrome
5	Metabolic-associated fatty liver disease	Nonalcoholic fatty liver disease; liver failure; liver fibrosis; medical examinations; liver diseases nonalcoholic fatty liver disease; metabolic-associated fatty liver disease; diet quality; coronary artery disease; fibrosis marker
6	Natural history	Nonalcoholic fatty liver disease; nonalcoholic steatohepatitis; FGF-based therapeutics; fibroblast growth factors; ketone bodies cardiovascular disease; hepatic fibrosis; myocardial energetic efficiency; fib-4 index; cardiac energetics
7	Cohort study	Nonalcoholic fatty liver disease; fatty liver index; cardiometabolic risk; sub-Saharan Africa; HDL-c ratio diabetes mellitus; cardiometabolic risk factors; liver fibrosis; fatty liver; metabolic diseases

FGF = fibroblast growth factor, HDL-c = high-density lipoprotein cholesterol, NAFLD = nonalcoholic fatty liver disease.

#ALT, #metabolic syndrome, #insulin resistance, and #diabetes mellitus have remained prominent research topics throughout the entire timeline. Over the years, researchers have engaged in extensive discussions and contributed significantly to understanding the correlation between MASLD/NAFLD and CVD through research on these topics. The emergence of new keyword clusters, such as #oxidative stress, #gut microbiota, #adipose tissue inflammation, #weekend catch-up sleep, #vegetarian diet, and #areca reflects a shift in research focus in MASLD/NAFLD and CVD research. In addition to #oxidative stress, #gut microbiota, and #adipose tissue inflammation discussed previously, healthy lifestyle choices, fibroblast growth factor (FGF)-based therapy, and gene polymorphisms have gradually emerged as new research hotspots.

FGF-based therapy is a therapeutic strategy that harnesses FGF and its associated signaling pathways to address diseases or facilitate tissue repair and regeneration. These therapies have diverse applications, ranging from treating tissue injuries and promoting healing to addressing CVDs, neurological disorders, metabolic conditions, and more.^[66–68]^ Investigating the relationship between gene polymorphisms in MASLD/NAFLD and CVD enhances our understanding of disease pathogenesis and individual variations. This exploration aids in identifying novel therapeutic targets and devising personalized treatment approaches. For instance, certain gene polymorphisms are closely linked to obesity-related genes (such as *FTO*), genes regulating lipid metabolism (such as patatin-like phospholipase domain-containing 3 gene [*PNPLA3*]), and inflammatory mediator genes (such as *IL-6*), influencing the onset and progression of MASLD/NAFLD. In addition, susceptibility genes for CVD (such as *APOE*, and low-density lipoprotein receptor) are associated with elevated risks of cardiovascular complications among patients with MASLD/NAFLD.^[69–76]^ These insights offer novel perspectives on diagnosing, treating, and preventing MASLD/NAFLD and related cardiovascular disorders.

## 4. Future perspectives

### 4.1. Trends in research on MASLD/NAFLD and CVD

This study examined the relevant literature on the association between MASLD/NAFLD and CVD from 2006 to 2024 and predicted future research trends based on the analysis results. Research efforts focusing on the association between MASLD/NAFLD and CVD originated in 2006, indicating the inception of this research direction. Building upon a robust foundation established by previous studies, currently, research on the association between MASLD/NAFLD and CVD has progressively evolved into a multidisciplinary field. Although classic themes, such as ALT, MetS, and insulin resistance, have consistently remained popular topics of discussion, novel themes, including inflammation, diet and lifestyle management, emerging therapies, and the involvement of intestinal microbiota, have emerged. These developments epitomize the most recent trends and directions in research endeavors that delve into the intricate association between MASLD/NAFLD and CVD.

### 4.2. Exploration of new themes

#### 4.2.1. #Gut microbiota

The gut microbiota comprise a diverse array of bacteria, fungi, viruses, and other microorganisms essential for various physiological functions within the human body. These functions include aiding in food digestion, synthesizing vitamins, regulating the immune system, and maintaining intestinal health.^[77,78]^ Furthermore, research has established connections between gut microbes and the development and progression of CVD, metabolic disorders, and other ailments. Animal models have demonstrated that specific microbes or shared microbe-host metabolites can causally contribute to CVD development through the gut microbiome. Large-scale cohort studies have also identified disruptions in the gut microbiota composition of patients with CVD, supporting the potential of modulating this microbial community for preventing and treating CVD.^[79–81]^ In addition, investigations into the intestinal microbiota have revealed close associations between alterations in these microbial populations and the onset and progression of MASLD/NAFLD. Imbalances in gut microbiota proportions have been linked to the occurrence of fatty liver and severity of inflammation. Moreover, through production of metabolites, such as bile acids, gut microbiota can influence MASLD/NAFLD progression.^[82–84]^ Therefore, targeting the regulation of gut microbiota has shown promise for preventing and treating these diseases.

#### 4.2.2. # FGF-based therapy

FGF is a pivotal cytokine facilitating the proliferation and migration of endothelial progenitor cells (EPCs), thus fostering neovascularisation. Consequently, FGF-based therapy holds promise for cardiovascular regeneration by bolstering EPC function and abundance.^[84–87]^ Moreover, FGF augments EPC survival and functionality through the activation of diverse signaling pathways such as Akt and hypoxia-inducible factor 1-alpha. In addition, FGF1 has been shown to regulate myocardial development via the FGF receptor-protein kinase C signal transduction pathway, indicating its potential application in myocardial remodeling therapy.^[68,88]^ However, further investigations are required to confirm its safety profile and clinical efficacy. Presently, FGF analogues have demonstrated therapeutic efficacy in patients with NAFLD and clinical trials have made significant strides in this direction. These analogues show their capability to mitigate hepatic steatosis, inflammation, and fibrosis associated with NAFLD pathology. Specifically, FGF19 and FGF21 exhibit significant potential for treating NAFLD by effectively reducing hepatic lipid accumulation and attenuating steatosis and fibrosis progression within the liver parenchyma.^[89–91]^ However, a comprehensive evaluation of the safety profile and therapeutic efficacy of both FGF1 and FGF4 in animal models and human patients remains imperative.

#### 4.2.3. # Gene polymorphisms

Some gene polymorphisms, such as those in the leptin receptor (*LEPR*) and apolipoprotein C3 (*APOC3*) genes, have been associated with both MASLD/NAFLD and CVD. Variants in the *LEPR* gene, such as 3057G > A and Lys656Asn, may contribute to the development of MASLD/NAFLD by influencing lipid metabolism and insulin sensitivity.^[92–95]^ Similarly, variants in the *APOC3* gene are linked to an elevated risk of MASLD/NAFLD and CVD, affecting lipid metabolism and insulin sensitivity, among other biological factors relevant to MASLD/NAFLD and CVD.^[96–98]^

One of the most extensively studied gene variants associated with MASLD/NAFLD are the variants in the *PNPLA3* gene. The G allele of PNPLA3 is associated with a higher susceptibility to steatohepatitis, liver fibrosis, and HCC. Another significant gene variant in MASLD/NAFLD is the transmembrane 6 superfamily 2 gene that increases the risk of fatty liver disease and liver fibrosis while potentially reducing the risk of cardiovascular events.^[70,99–101]^

Moreover, certain common single nucleotide polymorphisms have been identified to elevate low-density lipoprotein cholesterol levels, thereby increasing the predisposition for developing CVD. These polygenic contributions from single nucleotide polymorphisms collectively constitute polygenic familial hypercholesterolemia, distinct from monogenic familial hypercholesterolemia caused by pathogenic variants in low-density lipoprotein receptor, APOB, or PCSK9 genes, which confer a higher risk of CVD.^[76,102–104]^

## 5. Conclusion

MASLD/NAFLD and CVD, both significant health concerns, have gained significant research attention over the years. The intricate relationship between MASLD/NAFLD and CVD is influenced by a myriad of factors. Initially, research focused on metabolic dysfunction, highlighting common metabolic alterations shared by both conditions. MASLD/NAFLD and CVD are frequently associated with the presence of a MetS characterized by obesity, insulin resistance, elevated cholesterol, and hypertension, all pivotal in disease pathogenesis. Recent studies have shed light on the inflammatory cascade initiated by adipocytes, resulting in adipocyte demise, fatty acid release, and the secretion of inflammatory mediators, thereby fostering hepatic inflammation and fibrosis. These disruptions in lipid metabolism and release of inflammatory mediators also impact endothelial function, precipitating CVD development. Moreover, the etiology of MASLD/NAFLD and CVD encompasses various genetic determinants, with specific gene polymorphisms correlating with heightened disease susceptibility. However, further investigation is warranted to elucidate the precise genetic underpinnings of this association.

This study presents a comprehensive analysis of MASLD/NAFLD and CVD literature spanning 2006 to 2024, using Citespace software to visually map significant research trends and track keyword evolution. However, the study has certain limitations. Bibliometric analysis involves statistical evaluation of published literature but does not enable systematic aggregation and statistical synthesis of study-level data as meta-analyses do, nor does it assess the articles quality of included studies or validate causal relationships between variables. In investigating the mechanistic associations between MASLD/NAFLD and CVD, bibliometric analysis can only identify statistical associations through term co-occurrence frequency and co-citation analysis, without distinguishing direct mechanistic linkages. However, bibliometric analysis offers the capability to visually highlight seminal articles within a research domain, enabling readers to prioritize critical literature. For researchers new to the field, it serves as an efficient tool for identifying pivotal keywords. This methodology generates knowledge maps that intuitively display research hotspots and evolutionary trajectories within a field. Furthermore, burst detection facilitate the identification of emerging research directions and tracking of disciplinary development trends.

In summary, MASLD/NAFLD serves as an independent risk factor for CVD, with the severity of liver disease further amplifying the risk of CVD-related events. Consequently, clinicians should perform CVD risk assessments in individuals with MASLD/NAFLD and provide recommendations for lifestyle modifications and pharmacological interventions targeting CVD risk factors. Personalized treatment strategies tailored to individual variability should be formulated. Key interrelated indicators, including ALT, MetS, gut microbiota, oxidative stress, and adipose tissue inflammation, strongly associated with both MASLD/NAFLD and CVD, should be integrated into cardiovascular risk screening protocols. Moreover, for MASLD/NAFLD patients with atherogenic dyslipidemia, statin therapy is recommended to mitigate CVD risk. In addition, preventive measures for CVD in MASLD/NAFLD populations should emphasize lifestyle adjustments, including smoking cessation, alcohol restriction, regular physical activity, and reduced intake of trans fatty acids and high-sugar diets. Additionally, targeting gut microbiota modulation through probiotics, prebiotics, or fecal microbiota transplantation holds significant therapeutic potential. Our present study offers invaluable insights for future inquiries into MASLD/NAFLD and CVD, fostering a deeper comprehension of their pathogenesis and offering innovative strategies for prevention and treatment.

## Acknowledgments

We are grateful to all study participants for their cooperation.

## Author contributions

**Conceptualization:** Qinyi Zhou.

**Data curation:** Qinyi Zhou, Wang Liu.

**Formal analysis:** Qinyi Zhou, Wang Liu.

**Funding acquisition:** Zhaobing Li, Xiaofeng Ma.

**Investigation:** Qinyi Zhou, Wang Liu, Xiaofeng Ma.

**Resources:** Dan Zhou, Yifang Zhang, Zhaobing Li, Zili Li, Xiaofeng Ma.

**Software:** Wang Liu, Dan Zhou, Yifang Zhang.

**Writing – original draft:** Qinyi Zhou, Wang Liu.

**Writing – review & editing:** Qinyi Zhou, Wang Liu, Xiaofeng Ma.
